# Rituximab Induced Pulmonary Edema Managed with Extracorporeal Life Support

**DOI:** 10.1155/2018/6039045

**Published:** 2018-01-17

**Authors:** Jacob R. Miller, Warren Isakow, Muhammad F. Masood, Patrick Aguilar, Kristen M. Sanfilippo, Keki R. Balsara, Akinobu Itoh

**Affiliations:** ^1^Division of Cardiothoracic Surgery, Washington University, St. Louis, MO, USA; ^2^Division of Pulmonary Critical Care, Washington University, St. Louis, MO, USA; ^3^Division of Hematology, Washington University, St. Louis, MO, USA

## Abstract

Though rare, rituximab has been reported to induce severe pulmonary edema. We describe the first report of ECLS utilization for this indication. A 31-year-old female with severe thrombotic thrombocytopenic purpura developed florid pulmonary edema after rituximab infusion. Despite advanced ventilatory settings, she developed severe respiratory acidosis and remained hypoxemic with a significant vasopressor requirement. Since her pulmonary insult was likely transient, ECLS was considered. Due to combined cardiorespiratory failure, she received support with peripheral venoarterial ECLS. During her ECLS course, she received daily plasmapheresis and high dose steroids. Her pulmonary function recovered and she was decannulated after 8 days. She was discharged after 23 days without residual sequelae.

## 1. Introduction

Rituximab, a monoclonal anti-CD20 antibody used in the treatment of hematologic and autoimmune diseases, though rare, has been reported to induce life-threatening pulmonary edema attributed to a large cytokine release [1, 2]. The management of severe noncardiogenic pulmonary edema with extracorporeal life support (ECLS) has been described [3]. Further, ECLS has been utilized for pulmonary edema secondary to cytokine release in the setting of transfusion-related acute lung injury [4] and tumor lysis syndrome [5]. We present here a case of acute florid pulmonary edema due to a rituximab infusion, the first reported successfully managed with ECLS. This case report was approved by the Washington University in St. Louis School of Medicine Internal Review Board with the patient's consent.

## 2. Case Presentation

This 31-year-old female suffered from thrombotic thrombocytopenic purpura (TTP) with multiple relapses requiring plasmapheresis, steroids, and rituximab. Two weeks prior to presentation, she was hospitalized for a TTP relapse with a platelet count of 17,000, hemoglobin of 7.9 g/dl, lactate dehydrogenase of 1,246 units/L, undetectable ADAMTS13 activity level, and ADAMTS13 inhibitor level greater than 8 inhibitor units. While admitted, she underwent seven rounds of plasmapheresis and was started on weekly low-dose rituximab (100 mg fixed dose) per clinical trial. She received two doses during admission, which she tolerated with only mild itching. At that time, she was receiving prednisone, 100 mg daily and premedication with diphenhydramine and acetaminophen. She presented 6 days after discharge for week three of low-dose rituximab, receiving the same premedications, during which she developed respiratory distress. The infusion was immediately stopped; however, she quickly deteriorated requiring intubation, producing copious white frothy sputum that required continuous suctioning. A chest radiograph confirmed severe bilateral pulmonary edema. Despite advanced ventilatory settings and inhaled nitric oxide (NO), she developed respiratory shock (pH: 7.19, pCO_2_: 63 mm Hg, pO_2_: 42 mm Hg). Her lactate peaked at 18.0 mmol/L and she became oliguric. A transthoracic echo (TTE) demonstrated grossly normal left ventricular (LV) and right ventricular (RV) function and ejection fraction (EF) of 70%, with pulmonary artery systolic pressures (PASP) of 36 mmHg. She became difficult to oxygenate requiring escalating ventilatory settings, pressure-control ventilation with a peak inspiratory pressure of 40 cm H_2_O, and a plateau pressure of 20 cm H_2_O giving a mean airway pressure of 26–30 cm H_2_O, with a fraction of inspired oxygen of 100%. Her acidosis, elevated intrathoracic pressure, and increased pulmonary vascular resistance led to a significant vasopressor requirement. This was confirmed with a central venous catheter which measured her central venous pressure at 28 mmHg. Serial bedside ultrasounds were performed which showed a deterioration in left ventricular function with poor ventricular filling. An esophageal Doppler was placed which showed a cardiac output of 2.1 L/m for a cardiac index of 1.0 L/min/m^2^ (body surface area: 2.2 m^2^).

As this insult was anticipated to be transient and reversible, venoarterial (VA) ECLS was initiated. After 3,000 units of intravenous heparin, percutaneous cannulation was performed with a 25-French multistage cannula in the femoral vein and a 17-French arterial cannula in the common femoral artery. In addition, a 5-French vascular sheath was inserted into the superficial femoral artery for distal limb perfusion. All cannulas were placed with ultrasound guidance. The ECLS circuit initially provided a flow of 2.3–2.7 L/min/m^2^ with a sweep gas flow rate of 8 L/min at 100% oxygen. Once the patient stabilized a repeat TTE was performed which showed severe LV dysfunction with an EF of 25% and PASP similar to the previous.

Vasopressors and NO were immediately weaned. With oliguria and pulmonary edema, continuous venovenous hemodialysis (CVVHD) was initiated. Despite a negative fluid balance, chest radiographs worsened until ECLS Day 3. By Day 5, the radiographs began to improve and she was able to be weaned to conventional pressure-control settings with 40% FiO_2_. At this time, an echocardiogram demonstrated normal cardiac function. She received daily plasmapheresis and high dose steroids, methylprednisolone 1 mg/kg. As her acidosis corrected, the sweep speed was decreased. On Day 6, ECLS weaning was initiated gradually. She was decannulated on Day 8. After decannulation her pulmonary status continued to improve and she was extubated the next day.

Her course was well tolerated, without major complications. After decannulation she did well and was discharged after a total hospital stay of 23 days. She did develop acute kidney injury (AKI) prior to ECLS cannulation, further exacerbating her pulmonary edema. She received CVVHD with aggressive volume removal, net 300 ml/hour with plasmapheresis. Her renal function gradually improved and returned to baseline prior to discharge. Additionally, diffuse oral mucosal bleeding began immediately after cannulation. Though no intervention was necessary, due to this and her low platelet count, she was not anticoagulated. While maintained on ECLS, the initial goal platelet count was greater than 20,000/mm^3^. However, once she developed oral mucosal bleeding, the goal was modified to greater than 40,000/mm^3^. She received 9 platelet transfusions during her ECLS course.

When cannulated for ECLS her ADAMTS13 activity level was undetectable and her ADAMTS13 inhibitor level was 4.4 inhibitor units. Her platelet count reached a nadir of 12,000/mm^3^. By ECLS Day 7 she improved to an ADAMTS13 activity level of 61% and an inhibitor level of 0.4 inhibitor units. In total, she received 13 rounds of plasmapheresis and was transitioned to 100 mg prednisone daily. Since she is no longer a candidate for rituximab and has a high probability of relapse, she has been referred for splenectomy.

## 3. Discussion

Rituximab decreases the production of antibodies to ADAMTS13, leading to increased ADAMTS13 activity and cleavage of the large von Willebrand Factor multimers, thus, preventing the multimer-induced platelet aggregation and microthrombi formation occurring with TTP [6]. Expansion of rituximab use has resulted in the reporting of rare adverse events in postmarketing surveillance [7]. Cytokine release syndrome (CRS) has been reported, though this typically occurs after the first infusion [2].

Initially, our patient had a broad differential diagnosis. Her rapid deterioration, frothy sputum, chest radiograph, and timing made pulmonary edema due to the rituximab infusion the most likely diagnosis. Rituximab induced pulmonary edema due to the fact that CRS has been previously reported and shown to be reversible [1]. Therefore, despite her grave condition, we anticipated that she had a potentially recoverable insult.

By the time ECLS was considered she required high levels of support, both respiratory and hemodynamic. At this time, the patient was on advanced ventilator settings with high peak inspiratory and mean airway pressures, which causes a decrease in left ventricular stroke volume and cardiac output [8]. Additionally, acidosis, metabolic or respiratory (our patient suffered from both), is known to lead to a decreased stroke volume due to the negative inotropic effects of the acidosis [9]. This likely resulted in her hemodynamic collapse, and, therefore, VA, rather than venovenous (VV) ECLS, was initiated. In this instance, the VA ECMO strategy allowed for decreased vasopressor requirements and helped to improve renal blood flow. However, if considered earlier, VV ECLS may have prevented her hemodynamic compromise by eliminating the respiratory acidosis, the elevated intrathoracic pressure and the increased pulmonary vascular resistance, and, therefore, the need for VA support.

As our patient's cardiac function improved such that she essentially had only respiratory failure, there was concern for differential hypoxia. As cardiac function recovered, due to her cannulation strategy, her cardiac output would consist of poorly oxygenated blood preferentially perfusing the coronaries and cerebral vessels. Further, a “dual circuit” can develop, such that deoxygenated blood recirculates from upper body (heart, brain, and upper extremities) through the superior vena cava (SVC), heart, lungs, and great vessels. A second circuit is established consisting of the descending aorta, lower body, inferior vena cava (IVC), and the ECLS circuit [10]. To monitor for this, our patient had bilateral radial arterial access with arterial blood gas analysis from each. Had they demonstrated a developing differential hypoxia, alternative cannulation strategies would have been necessary, possibly including advancing the IVC catheter into the SVC or placing additional venous or arterial cannulas [11]. Placement of a subclavian arterial cannula in addition to, or rather than, a femoral cannula also would have abolished this phenomenon.

While the literature remains inconclusive, some suggest significant differences in survival based on cannulation strategy. Interestingly, veno-venoarterial (VVA) ECLS is touted by some as having superior survival, to both VV and VA, in patients with a primary pulmonary pathology [12]. This cannulation strategy utilizes a second outflow cannula, returning oxygenated blood to both the arterial and the venous system. If venous return is insufficient to support the increase in outflow, a second venous return cannula can be used. Indeed, despite the lack of differential hypoxia, our patient may have benefited from VVA ECLS by improving the oxygenation which would allow for lung protective ventilator settings earlier. Additionally, the preoxygenated blood flowing through the pulmonary system would lead to decreased pulmonary vascular resistance and further increase preload.

Since her hemodynamics continuously improved after VA ECLS initiation, though considered, the decision was made to not pursue a cannula reconfiguration. In addition to cannula reconfiguration, alternative therapies directed at the treatment of CRS could have been considered, rather than plasmapheresis. Recent literature has shown some promise with the anti-IL-6R mAb tocilizumab [13]. Though reports of its utilization are sparse, with only one report describing its utilization in 9 pediatric patients, the results are encouraging [14].

As new therapies are developed for hematologic, oncologic, and autoimmune diseases, the possibility of severe pulmonary edema related to CRS may become more frequent. Here, we demonstrate the utility of ECLS as a temporizing mechanism, though note that further research is necessary to determine optimal cannulation strategies.

## Abbreviations

ECLS: Extracorporeal life support
TTP: Thrombotic thrombocytopenic purpura
TTE: Transthoracic echocardiogram
LV: Left ventricle
RV: Right ventricle
EF: Ejection fraction
PASP: Pulmonary artery systolic pressure
NO: Nitric oxide
VA: Venoarterial
CVVHD: Continuous venovenous hemodialysis
AKI: Acute kidney injury
CRS: Cytokine release syndrome
VV: Venovenous
VVA: Veno-venoarterial
SVC: Superior vena cava
IVC: Inferior vena cava.

## Data Access

Data for this case report is not available to preserve patient privacy. The identified data is available on request from the corresponding author.
